# Structured Methods for reproducible science

**DOI:** 10.15252/msb.20188556

**Published:** 2018-07-25

**Authors:** Maria Polychronidou

**Affiliations:** ^1^ EMBO Heidelberg Germany

## Abstract

Detailed and accurate documentation of the reagents, tools and methods used in a study is key for reproducible science. However, the information provided in the Materials and Methods section is not always sufficiently detailed to allow for the adoption of methodologies across laboratories. Substantial time and effort, as well as extensive correspondence with the authors of a published paper, is often required in order to obtain all the relevant information related to a particular technique. Even after following a trail of references that frequently lead to a paper published decades ago, it is sometimes impossible to find a sufficiently detailed description of a technique “performed as described before”.

As a first step towards resolving this long‐standing issue, we are introducing *Structured Methods*, a new format for the Materials and Methods in *Molecular Systems Biology* (Fig 1). This new format includes two sections. The first one is a *Reagents and Tools Table* listing key materials—including reagents, experimental models and software—and their source and identifiers. The aim of this Table is to offer the reader a quick overview of the materials used in the study and to provide easy access to the information related to these materials. In the second section, *Methods and Protocols,* the authors are given the option to describe their methods in a protocol‐like format with bullet points, instead of the commonly used condensed narrative. The protocol format allows inclusion of details that are usually part of a protocol but generally do not make it to the summarized Materials and Methods text, such as notes on “tricky steps” in the procedure. Using the protocol format is optional, but we hope that it will encourage authors to provide a more detailed documentation of their methods, allowing other laboratories to easily adopt them. In addition, using this format will simplify writing the Methods section, since the authors will not need to re‐write their already documented protocols into a condensed narrative. The possibility to re‐use and adapt descriptions of protocols from the authors’ previously published work is an additional advantage and is compatible with our editorial guidelines <http://msb.embopress.org/authorguide#materialsandmethods>.

**Figure 1:**
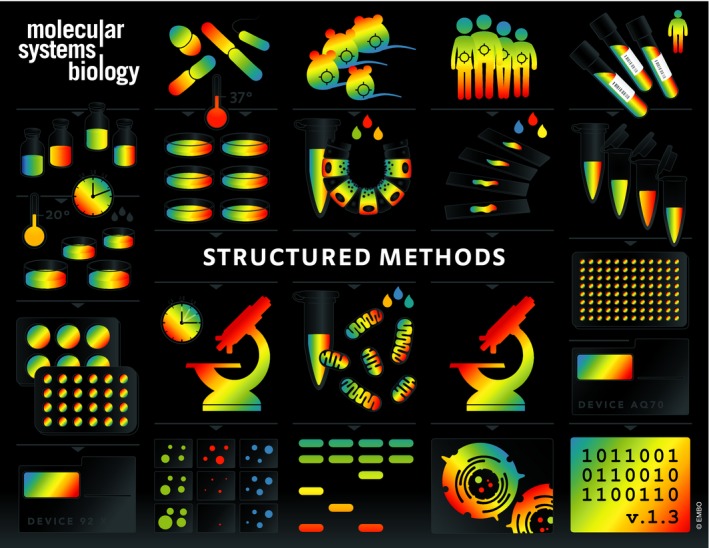
*Structured Methods* include a *Reagents and Tools Table* listing key materials and a *Methods and Protocols* section*,* where the authors are given the option to provide a detailed step‐by‐step documentation of their methods in a protocol‐like format

The precise description of reagents and procedures is especially important for allowing the widespread adoption of new methodologies, and therefore, we have decided to pilot *Structured Methods* in our Methods section. The first Method published using this format is the study by Wanker and colleagues (Trepte *et al*, 2018) describing a luminescence‐based two‐hybrid approach for quantitatively mapping protein–protein interactions. Until we formally implement *Structured Methods* across all article types, we also strongly encourage the authors of research Articles and Reports to use this format.

After the publication of a paper, the related protocols are frequently modified, optimized or adapted to new experimental conditions either by the authors themselves or by other researchers. To accommodate this, we are partnering with protocols.io (<http://protocols.io>), an open‐access platform for sharing experimental and computational protocols. Specifically, we encourage the authors who provide protocols in their *Molecular Systems Biology* paper to also submit them to protocols.io. This platform offers several benefits, including versioning the protocols when they are later modified, forking them to create derivatives similar to open‐source software, receiving feedback from other researchers who have used them in their laboratories, providing videos and images of experimental procedures in a protocol and grouping a protocol together with related ones for sharing with researchers working on similar topics. This way, the version of the protocol that was used in the published paper is firmly documented in the paper itself, but it can independently “evolve” on protocols.io. Authors who are already using protocols.io are encouraged to export their protocols from the platform for inclusion in their paper.

*Structured Methods* is one more step towards publishing reproducible science. Further options we are already exploring include using standardized identifiers for reagents, and tagging reagents and protocols to specific experiments in Figures in order to give the reader easy access to the Materials and Methods used to obtain specific results. We are also looking into developing automated text mining‐based methods that will allow the authors to extract reagent information from the text for filling the *Reagents and Tools Table* and to import laboratory protocols in a format that is compatible with the Materials and Methods section.

So far, we have received overwhelmingly positive feedback on *Structured Methods*, both from our Editorial Advisory Board and from the authors of the first Method papers using this format. We were excited to hear that these authors found the format straightforward to implement and felt that it will facilitate the adoption of their methodologies by other laboratories. We hope that our readers and reviewers will also find *Structured Methods* useful, and we welcome your feedback and suggestions.
